# Anatomy and Development of the Mammalian External Auditory Canal: Implications for Understanding Canal Disease and Deformity

**DOI:** 10.3389/fcell.2020.617354

**Published:** 2021-01-08

**Authors:** Mona Mozaffari, Robert Nash, Abigail S. Tucker

**Affiliations:** ^1^Centre for Craniofacial and Regenerative Biology, King’s College London, Guy’s Hospital, London, United Kingdom; ^2^Department of Paediatric Otolaryngology, Cochlear Implants, Great Ormond Street Hospital for Children NHS Trust, London, United Kingdom

**Keywords:** hearing, deafness, external ear, ear canal, ear deformities, congenital

## Abstract

The mammalian ear is made up of three parts (the outer, middle, and inner ear), which work together to transmit sound waves into neuronal signals perceived by our auditory cortex as sound. This review focuses on the often-neglected outer ear, specifically the external auditory meatus (EAM), or ear canal. Within our complex hearing pathway, the ear canal is responsible for funneling sound waves toward the tympanic membrane (ear drum) and into the middle ear, and as such is a physical link between the tympanic membrane and the outside world. Unique anatomical adaptations, such as its migrating epithelium and cerumen glands, equip the ear canal for its function as both a conduit and a cul-de-sac. Defects in development, or later blockages in the canal, lead to congenital or acquired conductive hearing loss. Recent studies have built on decades-old knowledge of ear canal development and suggest a novel multi-stage, complex and integrated system of development, helping to explain the mechanisms underlying congenital canal atresia and stenosis. Here we review our current understanding of ear canal development; how this biological lumen is made; what determines its location; and how its structure is maintained throughout life. Together this knowledge allows clinical questions to be approached from a developmental biology perspective.

## Introduction

**FIGURE 1:**
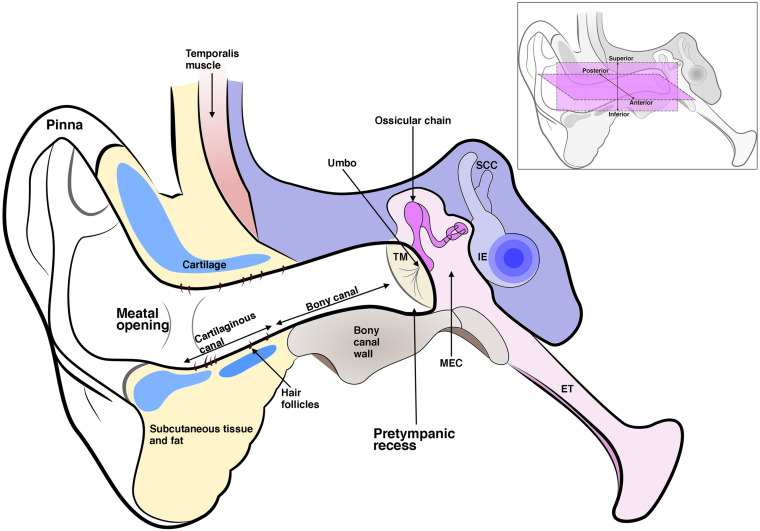
Schematic diagram of the three-part ear; the external ear, which includes the pinna and ear canal; the middle ear which includes the tympanic membrane (TM), middle ear cavity (MEC), and the ossicles. The malleus inserts into the TM at the umbo. The middle ear connects to the nasopharynx *via* the eustachian tube (ET). The inner ear (IE) includes the cochlea and semi-circular canals. The ear canal is enclosed in its inner two-thirds by a bony wall and its outer third by a cartilaginous wall, which is lined by hair-bearing skin. The ear canal lumen varies in shape but typically has a pretympanic recess and meatal opening. Inset shows anterior, posterior, superior, and inferior axes as referred to in the text.

Hearing places us within our external environment, allowing us to experience a multi-dimensional world, to listen and to communicate. Sound is essentially a series of pressure waves in our airborne environment. That we can “hear” these waveforms involves a complex array of neurophysiological mechanisms which begin at the outer ear and end at the auditory cortex. The mammalian ear is a crucial and fascinating sensory organ formed from the integration of three parts (Figure 1). The pinna or auricle directs sound waves into the external auditory Meatus (EAM), which then funnels sound waves toward the ear drum or tympanic membrane (TM), causing it to displace and move the ossicular chain of bones in the air-filled middle ear. The middle ear ossicles connect the TM to the much smaller oval window of the inner ear. This intricate leverage mechanism corrects the impedance mismatch between gas and liquid allowing airborne sound waves to move hair cells in the fluid-filled cochlea, generating neural signals that are transmitted to the auditory cortex *via* the cochlear nerve.

Hearing loss is most commonly caused due to pathology in the inner ear, referred to as sensorineural hearing loss, which consequently receives the lion’s share of scientific research (Cunningham and Tucci, 2017). However, there are limited options for treating sensorineural hearing loss with many potential treatments in the experimental phase (Devare et al., 2018). Currently, the clinician’s arsenal is best equipped to treat middle ear disease, which consequently receives the lion’s share of clinical attention (Cunningham and Tucci, 2017). This leaves the outer ear, which is also indispensable for hearing, but is comparatively overlooked. Pathologies of the ear canal can cause hearing loss, recurrent infection, and complications including cranial nerve palsy and intracranial sepsis (Ostrowski and Wiet, 1996). Management of hearing loss in this part of the ear may be particularly challenging, as the interaction between the anatomy and physiology of the canal, and the pathologies that affect it, may complicate the two most commonly used techniques of hearing rehabilitation – hearing aids and hearing restoration surgery.

In this review we focus in on the ear canal or EAM. We discuss the anatomy of the canal and the epithelial dynamics involved in canal homeostasis. We then turn to the congenital and acquired defects of the canal, and how they are currently treated, before investigating canal development. As anatomical and molecular details are added to our understanding of ear development, compelling questions are beginning to emerge. What factors determine the development of the ear canal’s specialized epithelium? And how does the canal integrate with the middle ear to form a functioning structure?

## Ear Canal Anatomy and Function

With certain exceptions, the EAM is similar across all mammals; a deep-set structure on either side of the skull ending in an ear drum cul-de-sac medially and opening to a pinna laterally (Figure 1). In adult humans, the EAM is typically 25 mm in length and 8 mm in diameter (Areias et al., 2016). Rather than a horizontal tube its shape is that of a soft sigmoid, tilted to face downward and inward as it projects medially (van Spronsen et al., 2014). This curved path varies between individuals (van Spronsen et al., 2014). Finite element modeling of the ear has highlighted the importance of a patent EAM in hearing higher frequency sound (which is crucial for speech recognition) (Areias et al., 2016). Lower frequencies with wavelengths larger than the canal itself are not affected by variations in canal diameter, however, as sound waves concertina at higher frequency, variations in canal dimension have a discernible effect on sound transmission. In particular, the segment of canal closest to the TM plays an important part in high frequency sound transmission (Areias et al., 2016). Variation in this pretympanic area, namely a deeper pretympanic recess, therefore can influence acquisition of high frequency sound, and has also been linked to increased susceptibility to chronic otitis externa (van Spronsen et al., 2014). Such biomechanical modeling at a patient-level would provide valuable information in understanding stenotic canal disease and measuring treatment outcomes.

**FIGURE 2:**
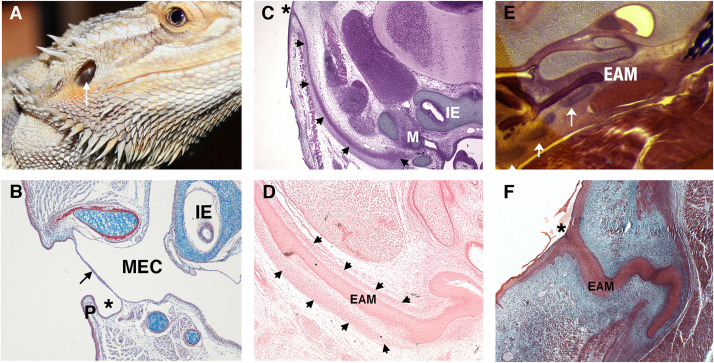
Variations in the non-mammalian ear canal. **(A)** Bearded dragon (*Pogona vitticeps*) with superficial tympanic membrane outlined by arrow. **(B–F)** Histology sections. **(B)** Sagittal trichrome section of gecko ear showing a shallow ear canal (star) and superficial tympanic membrane (arrow). **(C,D)** Developing platypus (*Ornithorhynchus anatinus*) EAM **(C)** Frontal section of platypus ear at 2 days post hatching (2 p.h.). The developing ear canal (arrows) is long extending from extending form the top of the head to the malleus. There is no pinna at the canal opening (marked by *). **(D)** Frontal section of developing ear canal in 10 p.h. day platypus. The EAM develops in a sheath of condensed mesenchyme (arrows). **(E,F)** Developing echidna (*Tachyglossus aculeatus*) EAM **(E)** Horizontal section through the EAM of 55–65 p.h. (arrows) days echidna demonstrating cartilage support (arrows) around the developing EAM. **(F)** Horizontal trichrome section through EAM of an echidna at 18 p.h. days showing a tortuous path to the opening (star). P, Pinna; MEC, middle ear cavity; IE, inner ear; EAM, external auditory meatus.

In contrast to the deep canal observed in most mammals, in sauropsids (reptiles and birds) and amphibia, the ear canal is typically shallow or non-existent (Figures 2A,B). Indeed, we can consider a deep canal to be a specific mammalian trait. It has been proposed that a cavitated middle ear and tympanic membrane evolved multiple times in land vertebrates (tetrapods), therefore the deep mammalian ear is not homologous to the shallow ear canal of other land vertebrates (Tucker, 2017). A deep canal is observed in both therian mammals (placentals and marsupials) and monotremes (egg laying platypus and echidna), however, monotremes do not have pinna, suggesting that this was a later therian adaptation as monotremes diverged from other mammals relatively early in evolutionary time (Figures 2C,F; Manger et al., 1998). The deep canal means that the mammalian tympanic membrane is protected within the head and therefore potentially at less risk of damage. Despite this, damage to the mammalian tympanic membrane is fairly common, with perforations being caused by loud noises, bangs to the head and from middle ear infections (Mozaffari et al., 2020). A deep canal also provides a means for funneling and thereby focusing sound waves, as discussed above.

Depending on the position of the middle ear with respect to the exit position of the canal, the relative length of the canal can vary across mammals. For example, in the platypus the ear canal exits high on the head, next to the eye, so that both the eye and ear are not submerged while the animal is swimming (Manger et al., 1998). This results in the development of a very long canal (Figure 2C). How the developing ear canal reaches the middle ear, finding its way to varying exit sites in different species, remains a largely unanswered question.

A deep-set lumen requires structural support. In humans, the inner two-thirds of the EAM is bony (typically 16 mm in length in the human adult) and the outer third is cartilaginous (around 8 mm in length), encasing the canal with a semi-rigid wall (Standrig et al., 2016). How the mammalian ear canal evolved remains an intriguing question. Did pinna cartilage elongate inward to meet the bony canal or did the cartilaginous canal evolve separately from the pinna? The latter appears more likely as monotremes have no pinna but still have a cartilaginous support that forms around the developing EAM (Figures 2D,E; Anthwal et al., 2020). Cartilage support for the EAM, therefore, likely predates pinna evolution.

The newborn human ear canal is soft and rudimentary along its length (Abdel-Aziz, 2013). The osseous canal is not evident during embryonic development so appears to develop postnatally (Ikari et al., 2013). The specifics of its development, however, remain vague. It is not yet known whether the tympanic ring, which is a membranous bone, extends posterolaterally to take part in creation of the osseous canal, or whether the osseous canal forms by endochondral ossification of an initially fibrocartilaginous scaffold. Both scenarios are described in textbooks and articles, however, there are as yet no published studies demonstrating the process (Abdel-Aziz, 2013; Standrig et al., 2016). The cartilaginous canal appears prior to and apparently independent of the osseous canal (Ikari et al., 2013). From around 15 gestational weeks, a chain of bar like cartilages form inferiorly between the tragus and helix. The anterior cartilage of the tragus and the posterior cartilage of the helix develop early and are consistent in shape; ring and plate-like, respectively (Ikari et al., 2013). Rather than fibrocartilage, glial fibrillary acid protein (GFAP) positive elastic cartilage makes up the outer cartilaginous canal (Ikari et al., 2013). The cartilages transform morphology *via* intermediate circular and triangle shapes to become H-shaped cartilage wrapping their arms upward around the developing EAM epithelium (Ikari et al., 2013). Interestingly, these bars of cartilage appear to develop along a fascial condensation linking Reichert’s cartilage and the tragus (Ikari et al., 2013). No evidence of a corresponding fascial plane extending out from Meckel’s cartilage has been described that would potentially precede a superior wall (Figure 1 insert). Unlike the anterior canal, the posterosuperior canal is usually deficient of cartilage in the adult (Standrig et al., 2016). There is significant individual variation in canal shape, which is perhaps not surprising given the complex of irregular shaped cartilages that interlink to form the cartilaginous ear canal (Bartel-Friedrich and Wulke, 2007). Clinically, this variability appears to be of little consequence. Inevitable gaps between the jigsaw of connecting cartilages does, however, provide a likely explanation for the etiology of congenital auricular pseudocysts that can occur in multiples between the perichondrium and the skin of the external ear, requiring drainage and pinna reconstruction (Secor et al., 1999).

The lining of the ear canal is continuous with skin covering the pinna laterally and the outer layer of the tympanic membrane medially (Mallo, 2000). Being an anatomical cul-de-sac ending at the TM, the ear canal is lined with specialized skin capable of migrating, such that it can self-clean with the epidermal layer sloughing off and moving outward – as opposed to upward in skin elsewhere (Alberti, 1964; Fuchs and Horsley, 2008). A failure of this self-cleaning mechanism leads to the build-up of sloughed off keratinocytes in the canal, causing hearing impairment from blockage and localized tissue damage from chronic inflammation (Naiberg et al., 1984). Indeed, failure to remove ear canal skin is also observed in keratosis obturans and ear canal cholesteatoma, which are uncommon but well recognized diseases of the external ear discussed further below (Naiberg et al., 1984; Persaud et al., 2004).

Experiments tracing the movement of ink-tattooed ear canal skin cells in mammals (including humans) show these labeled cells moving laterally, out of the canal over time (Alberti, 1964; Michaels and Soucek, 1991). This movement occurs roughly at the rate of fingernail growth with anterior canal skin demonstrating the fastest migration (Alberti, 1964). Does this migratory pattern have an epicenter at the tympanic membrane? Dated dye experiments suggest a radial pattern of epidermal cell migration spoking away from the umbo, at the center of the TM (Michaels and Soucek, 1990). More recent experiments using BrdU to chase and label proliferating cells suggest a more involved and dynamic source of stem and progenitor cells that migrate in different directions in different parts of the TM (Knutsson et al., 2011; Chari et al., 2018). Frumm et al. add further detail to the migratory nature of the keratinocytes in the tympanic membrane’s outer layer. Using a combination of lineage tracing, live imaging and single cell sequencing, they demonstrate that the TM epidermis has distinct stem cell and committed progenitor regions, located close to supporting mesenchyme (the manubrium and annulus respectively). The progeny of these cells migrate out across the TM, maintaining a thin vibratory surface (Frumm et al., 2020).

There are distinctions in the morphology of the skin that covers the bony canal versus the skin that covers the cartilaginous canal in humans. The former is thin and flush against the canal wall bone, whilst the latter is thickened with a spongey subcutaneous layer possessing modified sebaceous glands called cerumen glands. The bony canal skin, does not possess ceruminous glands or hair follicles (Standrig et al., 2016). Our observations of human cranial skin verses ear canal skin development suggest the latter develops precociously in relation to skin (Fons et al., 2020). This has interesting implications for experimental comparisons drawn between ear canal skin and skin elsewhere, as well as clinical implications, where skin from the limbs is used to reconstruct the canal lumen. If a different developmental timeline leads to different skin biology, transplanting skin into a reconstructed ear canal may not be the ideal graft tissue.

The cerumen glands are responsible for the production of ear wax. Serous secretions and sebum from the cerumen glands mix with sloughed keratinized squames. A natural lubricant, ear wax aids the self-cleaning function of the ear canal, and is thought to have antimicrobial properties (Shokry and Filho, 2017). Interestingly, a little like tongue rolling ability, ear wax type is a dimorphic trait. Whether your wax is wet or dry is determined by one single-nucleotide polymorphism (SNP) of a single gene (ABCC11) (Toyoda et al., 2009). Patients with dry wax, which lacks oily components, are at higher risk of suffering from cerumen impaction. Impacted ear wax is the single most common ear canal ailment to affect patients and may require regular removal, especially in those using hearing aids (Schwartz et al., 2017). The human ABCC11 gene has no orthologous gene in mammals, except for primates. In mice, cerumen production is from a single large gland, the glandula ceruminosa, which opens into a single duct near the TM (Gruneberg, 1971). Layout of glands and secretion type is likely to be species specific and relate to size and position of the canal. In mice, this gland is present from E15 (Gruneberg, 1971). Human ceruminous glands are noted at around 19 gestational weeks in published literature (Perry and Shelley, 1955). It is likely that ceruminous glands evolved from modified sebaceous glands, as like sebaceous glands these are holocrine glands, were the cells burst to release their content (Niemann and Horsley, 2012). Sebaceous glands fail to develop normally in patients with hypohidrotic ectodermal dysplasia (XLHED), suggesting involvement of the ectodysplasin (EDA) receptor signaling pathway in the development of sebaceous glands (del-Pozo et al., 2019). Similarly, Eda mutant mice have defects in sebaceous glands, with addition of Eda stimulants able to rescue the defect (Kowalczyk-Quintas and Schneider, 2014). Meibomian glands, another example of a modified sebaceous gland, also fail to develop in rodent models carrying hypomorphic mutations in the Edar signaling pathway consistent with an XLHED phenotype, with increased Edar signaling leading to larger meibomian glands (Chang et al., 2009). Recently Edar has been shown to be expressed in developing rat cerumen glands, highlighting a conserved role for this pathway in these holocrine glands (del-Pozo et al., 2019). In addition to the Eda pathway, Wnt and hedgehog signaling have been shown to play a role in skin sebaceous gland development and therefore may have a role also in cerumen gland development (Allen et al., 2003; Niemann et al., 2003). Further study into the developmental mechanisms of these unique glands would certainly be revealing.

## Development of the Ear Canal and How It Reaches Its Target

**FIGURE 3:**
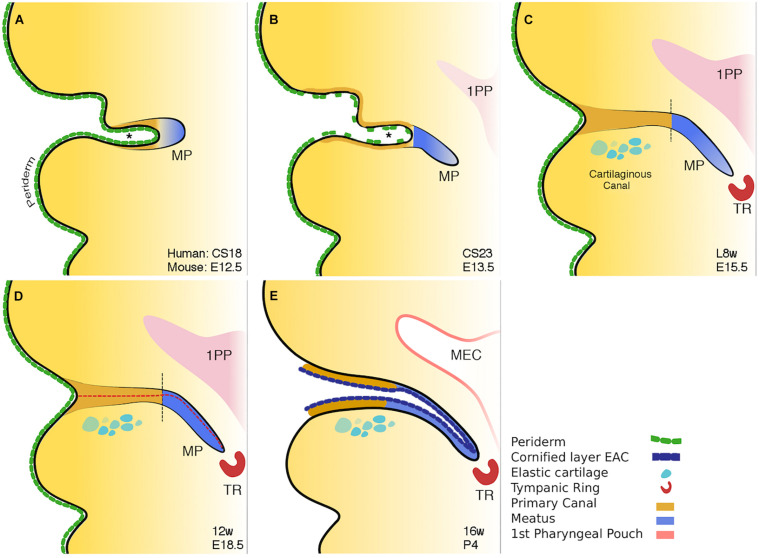
Schematic of mammalian ear canal development. **(A)** Invagination of the primary canal (marked by *) with a meatal plug at its tip. **(B)** Extension of the meatus toward the forming middle ear, driven by a potential signal from the tympanic ring. At the same time selective loss of periderm is observed in the primary canal caused by apoptosis. **(C)** Closure of the primary canal (following loss of periderm layer) and further extension of the meatal plug to reach the tympanic ring. **(D)** Loricrin expression (red dashed line) marks the site of opening of the canal. **(E)** Following keratinocyte differentiation, the whole external ear canal opens, with the upper/rostral wall of the meatal plate forming the outer surface of the tympanic membrane. Vertical black dashed lines in **C** and **D** between brown and blue regions represent the two distinct parts of the ear canal. Schematic taken from Fons et al. (2020).

The ear canal develops in two parts, the outer part or primary canal, and the inner part or meatal plug (Fons et al., 2020; Figure 3). At around Carnegie stage 17 (6 weeks post conception and equivalent to E12.0 in mice), the primary canal begins to form either by the first cleft deepening or as a new invagination within the 1st arch. Supporting the latter scenario is a recent study unexpectedly suggesting the murine ear canal forms entirely within the first arch (Minoux et al., 2013). This is unexpected because it had previously been assumed that the ear canal forms within the first cleft (Grevellec and Tucker, 2010). The meatal plug then forms as a plug of cells adjacent to this cleft. (Michaels and Soucek, 1991). This plug of cells will continue to extend inward into the surrounding mesenchyme becoming a finger-like projection pointing to the developing middle ear cavity (MEC) (Figure 3B; Nishimura and Kumoi, 2017). Temporal and situational differences in cell proliferation appear to influence the extension and directional growth of the meatal plug as it begins to develop (Fons et al., 2020). Both human and mouse display a distinctive duality in EAM development: the outer, primary canal (which will become the cartilaginous canal) develops differently to the inner, meatal plug (which will become the osseous canal) (Fons et al., 2020). Initially open, the primary canal closes for some time before opening again along with the meatal plug, to form one long lumen (Fons et al., 2020) (Figures 3C,E). This closure has been linked to the removal of periderm (Fons et al., 2020). In mouse embryos, a superficial layer of periderm, denoted by keratin 8 staining, acts as a non-stick Teflon layer to keep the primary canal open in early development (Fons et al., 2020).

Programmed cell death, or apoptosis, is a ubiquitous event in development. Its role in ear canal development has been previously alluded to in experiments noting the emergence of TUNEL-positive cells at E15.5 in mice with the surprising lack of them around the time of canal opening, up to 12 days postnatally (Nishizaki et al., 1998). Inhibiting apoptosis in culture and staining K8 positive peridermal cells demonstrated that it is the apoptotic death of the periderm that allows the primary canal to close at around E15.5 in mice (Fons et al., 2020). Indeed, in *Grhl3* null mice with deficient periderm, the primary canal closed prematurely, providing a hopeful avenue for further research into the etiopathology of congenital aural atresia (CAA) (Fons et al., 2020).

The canal finally opens by the creation of a central lumen (Figure 3E). This occurs at around 16 weeks in human fetal samples, and is preceded by a precocious program of differentiation, ahead of developing skin elsewhere, and involves the apoptotic cell death of the cornified loricrin-positive layer of squamous epithelium (Figure 3D). The complex and multistage development of the ear canal may account for the variability, both in form and occurrence, of CAA.

As with much of craniofacial development, the EAC is in essence an epithelial structure taking shape in relation to, and influenced by, its surrounding structures. How does the developing ear canal find its path to the middle ear cavity? Current knowledge tells us some detail about these relative relations and signaling pathways, putting the tympanic ring in a lead role. When tympanic ring formation is disrupted using retinoic acid, the EAC also fails to form (Mallo and Gridley, 1996). And when the tympanic ring is duplicated in *Hoxa2* null mutant mice, a duplication of the EAC is also noted, as the second arch transforms to a first arch fate (Rijli et al., 1993). In keeping with this phenotype, overexpression of *Hoxa2* in the first arch neural crest leads to a duplication of the pinna and loss of the EAM (Minoux et al., 2013). Additionally, *Prx1*, *Gas1*, *Tshirt (Tsh)*, and *Goosecoid (Gsc)* have all been shown to cause EAC defects in the presence of hypoplastic or absent tympanic rings (Rivera-Perez et al., 1995; Mallo, 2001; Seppala et al., 2007; Feenstra et al., 2011). Whilst EAC defects are observed in mouse mutants of *Prx1* and *Gas1*, *TSH*, and *GSC* have been corroborated in human genome wide sequencing studies also (Feenstra et al., 2007). Interestingly, mutations in *Gsc* and *Prx1* also cause hypomorphism of the malleal manubrium - an essential link in sound transduction from outside world to cochlea. Experiments in knock out mice as well as associative data corroborating human CT scans and intra-operative findings, indicate an essential role for the EAC in the induction and proper positioning of the manubrium within the eardrum (Mallo, 2000; Ishimoto et al., 2004). Not unlike an anatomical love triangle, these findings indicate that formation of the EAM depends on formation of the tympanic ring. In turn formation of the manubrium depends on formation of the EAM.

## Congenital Disease of the External Auditory Meatus

**FIGURE 4:**
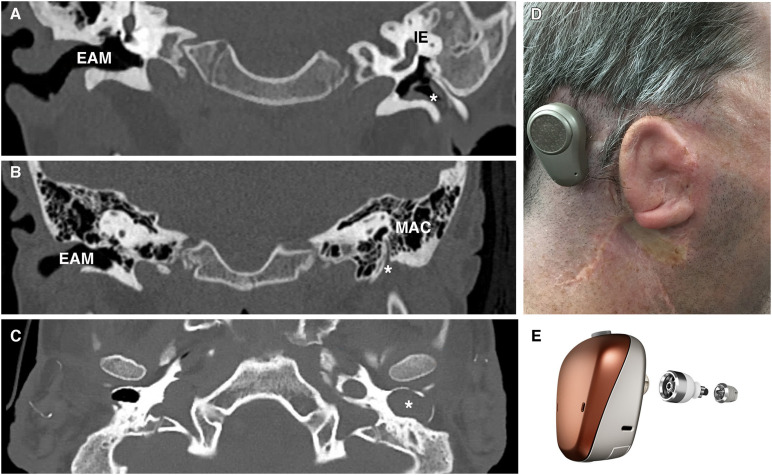
Patient photograph and scans of congenital aural atresia. **(A)** Congenital aural stenosis. Coronal CT scan shows a normal, patent right ear canal and a stenotic left ear canal marked by *. **(B)** Complete congenital aural atresia. Coronal CT scan shows a normal ear canal on the right and an atretic plate on the left with no ear canal. **(C)** Congenital aural stenosis and cholesteatoma. Axial CT scan of a patient with congenital aural stenosis and left-sided cholesteatoma. The bony ear canal has been widened and remodeled by the collecting keratin (marked by *). **(D)** Photograph of a patient with Treacher-Collins syndrome and complete aural atresia of the left ear canal. The patient has been fitted with an osseointegrated hearing device visible behind the pinna. **(E)** Example of an osseointegrated device. Computer-generated image of a BAHA Connect (Image courtesy of Cochlear Bone Anchored Solutions AB, ©2020). EAM, external auditory meatus; IE, inner ear; MAC, mastoid air cells.

Congenital aural atresia is a spectrum of defects affecting the EAM at birth. The EAM may fail to form partially, completely or be narrowed. Occurring in around 1 in 10,000 to 20,000 live births, CAA affects males more than females and is typically unilateral, involving the right side more commonly than the left (Abdel-Aziz, 2013). Over the years, a number of systems for classifying CAA have been proposed (Altmann, 1955; Schuknecht, 1989; Jahrsdoerfer et al., 1992). More typically, clinicians group CAA patients into complete atresia (complete obliteration of the canal usually in combination with a bony atretic plate), stenosis (in which there is a continuous lumen to an at least partial tympanic membrane) or partial atresia (variable presence of a canal lumen lateral to an atretic plate) (Figure 4; Tian-Yu and Bulstrode, 2019). Complete atresia and stenosis are much more common than partial atresia, which is rare (Nicholas and Kesser, 2013). Severity of canal atresia has been linked with an early arrest in EAM development, although this does not account for the striking difference in the incidence of complete verses partial atresia (Abdel-Aziz, 2013; Nicholas and Kesser, 2013). Recent studies described above, examining EAM development at a range of timepoints and using molecular cues suggest a more involved etiology at play, which may, for example, begin to explain the surprising rarity of partial in comparison to complete atresia (Fons et al., 2020).

## Genetic Basis of CAA

Congenital atresia is often associated with pinna and middle ear defects (Kountakis et al., 1995). Less frequently inner ear deformities and deformities of the stapes footplate are also present (Vrabec and Lin, 2010). Associations with other craniofacial abnormalities such as facial asymmetry and cleft lip/palate are also common (Abdel-Aziz, 2013). In some patients, these craniofacial defects are part of syndromic conditions such as Treacher-Collins, de Grouchy (chromosome 18q deletion), and Branchio-Oto-Renal syndrome (Figure 4D; Abdel-Aziz, 2013). Genetic analysis of syndromic patients can provide valuable genetic background to the etiology of both monogenic and syndromic CAA. For example, Genotype-phenotype mapping of chromosome 18q deletions (responsible for de Grouchy syndrome) has consistently shown the critical region for CAA in these patients to be 18q22.3 (Feenstra et al., 2007). Further delineating critical regions have highlighted candidate genes to investigate, with a loss of function mutation in the gene *TSHZ1*, known to be essential for murine middle ear development, being responsible for CAA both in isolated cases and in 18q deletion syndrome (Feenstra et al., 2011). A similar approach in a cohort of patients with the syndrome SAMS (short stature, auditory atresia, mandibular hypoplasia, and skeletal abnormalities) has identified mutations in the *Goosecoid (GSC)* gene as a causative factor (Parry et al., 2013). *GSC* is an important regulator of neural crest/mesoderm cell fate and has been shown to modulate tympanic ring development (Rivera-Perez et al., 1995). A canal defect in these patients may therefore be secondary to a tympanic ring defect, assigning a key signaling role to the tympanic ring (Mallo and Gridley, 1996).

Mutations in *HOXA2* have been characterized in a number of families with microtia (Alasti et al., 2008). Only one of these families, however, were noted to have additional EAM defects, with shortened and stenotic canals (Alasti et al., 2008). Mouse studies analyzing hypomorphic *Hoxa2* mutants with 45% the normal level of *Hoxa2*, and conditional mice, where loss was activated relatively late at E12.5, did not show any defects other than stapes and pinna deformity, respectively (Mallo and Gridley, 1996; Minoux et al., 2013). This is consistent with observations that *Hoxa2* requirement in craniofacial development is temporal (Santagati et al., 2005). It may be that low *Hoxa2* levels only effect EAM development in the early embryonic period by affecting arch fate, converting second arch to a first arch fate. That Alasti et al. report stenotic and shorted canals rather than completely atretic canals is interesting because it contradicts previous thinking that earlier mutations in EAM development lead to more severe anatomical defects (Abdel-Aziz, 2013; Nicholas and Kesser, 2013). It is likely that a more complex molecular basis is at play.

Genes discussed thus far play an indirect role in ear canal development, presumably due to their effect on the tympanic ring. *Foxi3*, in contrast, is likely involved in the development of the ear canal epithelium itself, making it a particularly interesting gene in the study of outer ear embryology (Tassano et al., 2015). Famously identified in hairless dogs, the *Foxi* family of genes are expressed in the ectoderm and play important roles in early arch patterning and thus early craniofacial development (Mukherjee et al., 2018). To date, mutations in *Foxi3* have been implicated in ear canal defects in mice, dog, and human, suggesting it plays a role in cases of canal atresia (Tassano et al., 2015). What is currently known about the role of *Foxi3* in EAC development is limited and associational. Unfortunately, *Foxi3* homozygous null mutants do not survive postnatally, and heterozygous mutants do not show a phenotype. A conditional mouse model would provide an intriguing avenue for further research, in particular into the mechanisms underlying the initiation of a meatal plug in early EAM development.

Given that CAA is a phenotype observed in multiple syndromes, it is not surprising that multiple genes have been associated with its etiopathology. The exciting next step will be to harness this information, using conditional knock outs or culture setups, to answer questions such as, what are the signaling pathways allowing the tympanic ring, ear canal, and manubrium to communicate?

## Treatment of Congenital Ear Canal Disease

Dividing canal atresia into complete, partial and stenotic is important to the clinician as it maps patients to key treatment considerations: hearing outcome, likelihood of cholesteatoma (erosive keratin-filled cysts that form when EAM skin cannot migrate out as normal) and the feasibility of achieving a self-cleaning ear should atresiaplasty be considered (Edfeldt and Strömbäck, 2015).

Esthetics aside, hearing loss is the main problem in complete atresia, as the absence of a lumen precludes the possibility of cholesteatoma forming. Despite significant advances in surgical technique and patient selection, atresiaplasty remains a difficult operation with typically poor outcomes and risk to the facial nerve, which is characteristically laterally placed, and therefore unexpectedly closer to the surgeon’s drill (Edfeldt and Strömbäck, 2015). Surgical atresiaplasty predates the use of bone anchored hearing aids (BAHAs), and it is still performed in healthcare settings with limited access to audiological services (Yellon, 2011). Complications include restenosis, recurrent keratinous debris build up, poor hearing outcomes (in one study as many as 93% of patients still required a hearing aid post-op) and facial palsy resulting from altered and variable anatomy of the facial nerve in CAA (Bauer et al., 1994). Once the neo-lumen is created, it is covered with skin grafts taken from elsewhere or pedicled from postauricular skin. Restenosis is prevented by performing a wide canalplasty, although this may have cosmetic implications. As there is no native canal skin to use, the neo-lumen has no capacity to clear desquamated debris, requiring regular aural microsuction (Li et al., 2016). Hearing outcomes are typically poor despite the opening of the canal and patients may still need a hearing augmentation device as well. Meatoplasty, where the stenotic canal is widened, is more frequently considered in cases of congenital aural stenosis. These patients carry a much greater risk of cholesteatoma to the presence of a narrow canal, which may inhibit epithelial migration (Cole and Jahrsdoerfer, 1990). Furthermore, native canal skin is often available for canal reconstruction reducing chances of post-operative complications, making surgery a more straightforward decision (Cole and Jahrsdoerfer, 1990).

Given the difficulties relating to surgical correction of ear canal atresia, bone conduction devices and auditory implants are often considered instead of atresiaplasty (Nadaraja et al., 2013). Bone conduction devices bypass the outer and middle ear by transmitting sound vibrations to the cochlea *via* the skull. Their connection to the skull may be temporary, using methods such as a headband or adhesive; or it may involve the placement of an auditory implant (Figure 4E). Auditory implants can be classified into percutaneous devices, active and passive transcutaneous devices, and middle ear implants. Patient factors, middle ear anatomy and clinician preference determines which devices can be offered (Kohan and Ghossaini, 2019). The option of implantable hearing aids is attractive, however, they require near to normal middle ear anatomy and thus are not suitable for many CAA patients.

In reality, in Europe and the United States, the majority of patients receive bone anchored hearing aids (Yellon, 2011). Whilst these provide good hearing rehab, they are visible, can cause skin irritation and infection and of course do not create a continuous hearing response (they are taken on and off), which is particularly important for spontaneous learning in young patients (Yellon, 2011). Therefore, a surgical approach which reconstructs the ear canal with few complications and without the need for further device-assisted hearing, is attractive. Recent research investigating epithelial dynamics in the developing ear canal may provide new avenues to explore the biological basis of atresiaplasty complications (Fons et al., 2020). For example, surgeons note the greater frequency of restenosis in the lateral reconstructed canal, which is cartilage-lined (Moon et al., 2012). As the bony and cartilaginous parts of the canal develop in different ways (primary canal verses an extending meatal plate), the development may explain these differences in response after surgery. It would therefore be interesting to study whether there is an inbuilt signaling difference in the outer and inner parts of the EAM that influences their response to injury. A better understanding of EAM development would also further clarify the variable anatomy in CAA, e.g., the route of the facial nerve, easing technical challenges faced by surgeons.

## Acquired Disease of the External Auditory Meatus

Acquired disease of the EAM includes of course the breadth of traumatic, infective and rarely neoplastic disease and its comprehensive review is beyond the scope of this review. These can all lead to a post-inflammatory canal atresia, sometimes referred to as stenosing otitis externa (Lavy and Fagan, 2000). Current treatment involves regular aural hygiene or meatoplasty to expand the canal opening. There is experimental evidence in Guinea pigs for the use of Mitomycin C (an inhibitor of fibroblastic activity) in preventing post-inflammatory canal stenosis (Yoon et al., 2010). Human studies, however, have had contradictory results and further controlled trials are needed to evaluate its potential role in preventing restenosis (Banthia and Selesnick, 2003; Battelino et al., 2005). Here, we focus on ear canal diseases that may be further understood through the lens of developmental biology.

Specific to the specialized biology of ear canal skin and its ability to migrate are two distinct diseases: keratosis obturans (KO) and ear canal cholesteatoma. KO is a rare disease in which squamous debris builds up in the ear canal, often leading to an expansion of canal diameter before presenting with acute pain. Current treatment is regular microsuction to maintain a patent ear canal (Persaud et al., 2004). As with healthy patients, the TM of individuals diagnosed with KO can be labeled with dye to observe the migration of the epidermal layer. One such study suggests that either delayed or disrupted epidermal migration may be the cause of KO (Corbridge et al., 1996). External ear canal cholesteatoma (EECC) is also rare and was for a long time grouped together with keratosis obturans as the same disease (Persaud et al., 2018). Whilst ear canal skin in KO is intact with layers of squames building up above it, in EECC there are focal ulcerations in canal skin and the organization of keratinous squames is random. Importantly there is bony necrosis with focal areas of deep sequestered bone (Persaud et al., 2018). As such, drawing a diagnostic distinction is important, as whilst KO can be managed conservatively, EECC typically requires surgical removal of all the cholesteatoma and reconstruction of the ear canal (Persaud et al., 2018). The etiopathology of EECC remains uncertain with disordered epithelial migration and/or cerumen gland dysfunction thought to play a role (Jackler et al., 2015). Localized differences in epithelial cell migration in the tympanic membrane have recently been proposed to underly propensity to middle ear cholesteatoma formation and may provide clues to the etiology of canal cholesteatoma also (Frumm et al., 2020). The underlying pathology of another category of cholesteatoma, congenital cholesteatoma of the middle ear, also remains controversial. Whilst current consensus rests with an epidermoid formation origin within the middle ear, another proposed theory suggests that an errant developing EAC extending beyond the tympanic ring and into the mastoid cavity may be at fault (Aimi, 1983). What determines epithelial behavior in the ear canal, driving it to focal erosion in EECC rather than circumferential upward layering into the lumen as in KO? Further understanding the epithelial dynamics that bestow EAM skin its migratory potential and its regenerative capacity will certainly begin to answer this question.

## Future Questions

Of the three-part mammalian ear, the external auditory canal receives arguably the least attention. Although this has not always been the case. Until the mid-twentieth century and the advent of hearing aids, an exaggerated external ear in the form of an ear trumpet was the most commonly used hearing device, emphasizing the valuable role of the external ear in hearing. Congenital disease of the EAM is thankfully rare but crucially important. Infancy is an irretrievable period in which to develop speech and language, which can determine the rest of a child’s life. Clarity and spontaneity of hearing, as well as decibels gained, at this age is critical. Thus, current treatment options, BAHAs or complication-prone canalplasties, leave room for improvement. Better understanding the mechanisms underlying EAM development will provide valuable knowledge toward improving treatment options both for congenital canal atresia and also treatment of acquired canal disease that involves epithelial dysfunction, such as keratosis obturans or canal cholesteatoma.

Recent research has pushed forward valuable but dated, largely histology-based, knowledge of EAC development and created new questions. How exactly does the ear canal open along its length, creating innermost a single-cell layer thin tympanic membrane, a thinly lined osseous canal and a spongier outer cartilaginous canal? What determines the different behavior of epithelium along the canal postnatally? During development, how do the different part of the complex mammalian ear communicate and eventually integrate? Answering these questions will pave the way to answering the clinical quandaries that affect patients’ lives. How to create a self-cleaning ear? How to recreate the external ear epithelium? Which pathways can be modified in the face of congenital ear disease? Looking to developmental biology for the answers will be key.

## Author Contributions

MM was responsible for conception and writing of the manuscript. AT contributed to the manuscript conception and reviewed the manuscript throughout the writing process. RN provided clinical images and reviewed clinical sections of the manuscript. All authors read and contributed to the manuscript and discussion.

## Conflict of Interest

The authors declare that the research was conducted in the absence of any commercial or financial relationships that could be construed as a potential conflict of interest.
